# How to quantify individuality in music performance? Studying artistic expression with averaging procedures

**DOI:** 10.3389/fpsyg.2013.00361

**Published:** 2013-06-19

**Authors:** Clemens Wöllner

**Affiliations:** Institute of Musicology and Music Education, University of BremenBremen, Germany

In artistic fields such as western music performance of the past 200 years, individuality is highly valued as a performer's expression of his or her aesthetic concepts. Yet characterizations of individual performance qualities have largely remained on a descriptive level. In this opinion article, it is argued that if researchers aim at quantifying individuality, then the only feasible approach is to determine the baseline from which individual performances diverge. Rather than using a computer-generated “deadpan performance” with no expressive features, this baseline should refer to average features comprising a number of different human performances in a given cultural context.

## Averages as prototypes

Research in cognitive psychology has shown that people prefer average features in visual and auditory modalities. Averaged human faces (Galton, 1878; Langlois and Roggman, 1990) and voice utterances (Bruckert et al., 2010) were rated as more attractive. Averaging procedures typically result in even, smooth visual displays or sounds that show no extremes in any feature. Psychological theories suggest that people construct mental prototypes based on a large number of individual objects or people they encounter in their lives—an idea already expressed by Kant (1790/1995) in *Critique of Judgment*. Averaged individual features can thus approximate mental prototypes within an epoch or culture. Displays with prototypical characteristics conform to people's expectations and enable more fluent processing, which in turn may cause a cognitive bias for averages (Rubenstein et al., 1999). Objects that are easily processed are thus often perceived to be more attractive. This bias resembles the enhanced ease of processing for repeatedly presented stimuli as demonstrated in the well-known mere exposure effect.

## Expressiveness indicates individuality

These advantages for averaged features stand in remarkable contrast to notions of artistic and musical individuality. Listeners typically do expect more from a concert or a recording than a smooth and even performance. In popular music genres, the sound of a singer's voice, of the instruments and the mix of audio tracks are often aimed at conveying a distinct, individual character (cf. Frith, 1998). In classical genres, emphasis is laid on subtle timing perturbations and fluctuations in dynamic intensity. Sudden delays or changes in intensity that do not conform to prototypical expectations may cause surprise and other emotional reactions (Huron, 2006) and reveal the individual performer's musical intentions. Research along these lines has for a long time studied expressive timing deviations from a non-expressive metronomic version. These timing deviations constitute an individual expressive microstructure (for an overview, see Clarke, 1995). Although it can be revealing to analyze the lengthening of note values in a final ritard for a number of different performers or for historical recordings during the course of the 20th century, no statements can be drawn about the *degree of individuality* in these performances. In other words, an expressive microstructure of a performance does not reveal *per se* whether the performance will be perceived as being individual. In earlier decades of the 20th century, for instance, musicians typically employed large *rubati* (Timmers, 2007) in accordance with listeners' expectations of that time, while nowadays these variations would not conform to prototypical listening expectations. Furthermore, timing deviations are to some degree also caused by human physiological constraints (Loehr and Palmer, 2009); performers are thus not able to render a perfect mechanical, metronomically exact performance. For these reasons, deviations from so-called deadpan renditions are no valid indicator of individuality.

## Perceptions of individuality may diverge from quality judgments and behavioral advantages

In contrast to many other forms of art, musical performances can be averaged according to the main quantifiable dimensions of duration, dynamic intensity, and pitch. MIDI technology allows for both relatively simple analysis of these parameters from a given set of individual performances as well as synthesis, which results in an averaged performance approximating mental prototypes. In a seminal study by Repp (1997), experienced listeners ranked the quality of artificial piano performances with averaged timing patterns higher as compared to actual performances with individual timing. This outcome was obtained both for student and professional pianists, some of their performances showing large deviations from the average timing pattern. At the same time, individuality of averaged performances was ranked lower. These results suggest that averaged musical performances are preferred in one dimension (quality) as prototypes, while on the other hand, they may be perceived as somewhat “dull” in comparison with some highly individual performances.

In a recent study, we asked whether quantitatively averaged point-light displays of orchestral conductors are perceived as prototypes and lead to advantages in behavioral and evaluative experimental tasks (Wöllner et al., 2012). While conductors shape musical performances according to their individual expressive intentions, they also need to organize the balance of the orchestral sound and synchronize the timing by means of gestures. In order to be recognizable to a large number of different musicians, there are thus constraints and limits to individuality. In our study, twelve orchestral conductors were recorded with a 3D motion capture system while they conducted typical four-beat measures with metronome-controlled timing. Based on the horizontal and vertical dimensions, averages were created and presented as point-light displays to participants in an experiment. Their task was to tap to the beat and to evaluate the conductors in terms of quality, clarity of beat, conventionality, and expressiveness. Our analyses revealed advantages for prototypes in action responses, which adds to previous research using perceptual judgments of attractiveness or quality. Participants' synchronization with averaged conducting displays was more consistent (reduced tapping variability) and more synchronous (smaller asynchronies) compared with displays of individual conductors. Kinematic analyses revealed reduced normalized jerk in averaged conducting, indicating smoother movements than for individual displays. Averages were also judged to be more conventional, which demonstrates that participants indeed perceived them as prototypes. Beat clarity of conducting gestures and quality, in contrast to Repp's (1997) findings, were not significantly higher for averaged compared with individual movements. Yet individual conductors were perceived to be more expressive. As a consequence, the predictability and smoothness of prototypical movements enhanced action responses, given that they were easier to perceive and process, while individual expressiveness was reduced. For fields with transitive gestures such as orchestral conducting, then, experienced individuals need to balance the functionality of their profession with the demand of conveying their distinct expressive intentions.

## Quantifying individuality

Apart from the above mentioned investigations of expressive timing deviations using MIDI technology, studies of musical individuality may focus more on musical timbre, pitch and intensity. Methods used by Bruckert et al. (2010) for acoustical morphing of the human voice could be employed to investigate individual musical timbres. Musicians are able to shape the timbre of certain instruments to some extent. Similarly, for singers as well as for instruments without fixed pitch such as many wind and string instruments, averaged deviations from notated pitch in equidistant temperament could be analyzed. The sharpening or flattening of tones may reveal certain expressive intentions of individual performers. Intensity can be measured with MIDI technology or by acoustical analyses. Studies have shown some dependencies between timing and intensity fluctuations (cf. Parncutt, 2003), and it would furthermore be revealing to analyze relationships with timbre and pitch in a systematic way.

When measuring the four musical dimensions of timing, intensity, timbre, and pitch to capture an individual musician's “fingerprint” of his or her performance, researchers may consider three relatively novel approaches in the field. First, as argued above, performances can be averaged according to these dimensions. In comparison to the analysis-by-synthesis approach that has been employed primarily for the study of the human voice (Sundberg, 2006), averaged performance dimensions are not artificially generated to produce a naturally sounding voice or instrument. Rather, distances to a synthesized performance based on actual renditions are used to estimate individuality. Averaging and synthesizing music may, as a caveat, result in the loss of some musical detail present in individual performances, and the averaged timbre may even sound unnatural, since the averaging procedure smoothes out extremes. It is also worth considering whether one dimension in question should be investigated while keeping the others constant. Repp (1997) only averaged timing patterns and compared them to individual performances while using the same timbre, intensity and pitches for all examples he presented to participants. Researchers can thus measure continuous deviations from an averaged performance in any dimension or combination of dimensions. Second, rather than only employing perceptual judgments, action-specific effects should be investigated. Synchronization studies offer a particularly valid research paradigm in the field of music. People may behave differently to stimuli that conform to their mental prototypes, even if they are not aware of these effects. Therefore, it is intriguing to combine perceptual and behavioral tasks for analyzing the dimensions that distinguish individual performances from others. Third, musical performers may take part in research studies both as musicians and listeners/observers of their own performances to investigate sense of agency for individual musical characteristics. In a study using motion capture of a Mendelssohn string symphony (Wöllner, 2012), orchestral conductors were able to identify point-light displays of their own conducting movements, while the corresponding short musical excerpts or point-light displays of gait did not contain sufficient cues for distinguishing their individual performances from those of other conductors.

## Conclusion

Individual artistic expression should be considered in the boundaries of a given cultural context. It can be defined as deviation from a prototypical exemplar within this context. Research suggests that averages composed of a number of individual objects or performances approximate people's mental prototypes. These prototypes have no universal validity, since cultural norms vary and change considerably across time even for fairly specific questions such as what is considered to be an appropriate performance of a musical piece. The curious research finding that prototypes are only preferred in some dimensions such as quality—while they are rated lower in important dimensions such as expressiveness—should be given more attention. It may well be that even for performing arts there are limits to individuality as soon as overall quality and mastery of a technical skill come into question. Finally, the development of individual performance manners should be addressed. A great deal of learning occurs implicitly by imitating influential others or by trying to reach the standard of a given prototype, and individual intentions need to be well balanced with cultural norms.
